# Effects of Selected Polysorbate and Sucrose Ester Emulsifiers on the Physicochemical Properties of Astaxanthin Nanodispersions 

**DOI:** 10.3390/molecules18010768

**Published:** 2013-01-09

**Authors:** Navideh Anarjan, Chin Ping Tan

**Affiliations:** 1 Department of Food Technology, Faculty of Food Science and Technology, Universiti Putra Malaysia, 43400 UPM Serdang, Selangor, Malaysia; E-Mail: anarjan@gmail.com; 2 Young Researchers and Elites Club, East Azarbaijan Science and Research Branch, Islamic Azad University, 15655/461, Tabriz, Iran

**Keywords:** nanodispersion, astaxanthin, emulsification-evaporation, emulsifier, polysorbates, sucrose esters

## Abstract

The effects of selected nonionic emulsifiers on the physicochemical characteristics of astaxanthin nanodispersions produced by an emulsification/evaporation technique were studied. The emulsifiers used were polysorbates (Polysorbate 20, Polysorbate 40, Polysorbate 60 and Polysorbate 80) and sucrose esters of fatty acids (sucrose laurate, palmitate, stearate and oleate). The mean particle diameters of the nanodispersions ranged from 70 nm to 150 nm, depending on the emulsifier used. In the prepared nanodispersions, the astaxanthin particle diameter decreased with increasing emulsifier hydrophilicity and decreasing carbon number of the fatty acid in the emulsifier structure. Astaxanthin nanodispersions with the smallest particle diameters were produced with Polysorbate 20 and sucrose laurate among the polysorbates and the sucrose esters, respectively. We also found that the Polysorbate 80- and sucrose oleate-stabilized nanodispersions had the highest astaxanthin losses (*i.e*., the lowest astaxanthin contents in the final products) among the nanodispersions. This work demonstrated the importance of emulsifier type in determining the physicochemical characteristics of astaxanthin nano-dispersions.

## 1. Introduction

Emulsifiers are surface-active substances that can be adsorbed at an oil-water interface and prevent the dispersed-phase droplets in emulsions from aggregating [1]. Small molecular emulsifiers are among the most frequently used emulsifiers in the food and pharmaceutical industries. These molecules can usually be rapidly adsorbed onto the phase interface to reduce the interfacial tension and prevent droplet coalescence during homogenization [2,3,4]. These emulsifiers vary widely in their ability to produce and stabilize emulsions depending on their physicochemical and molecular characteristics [3,4,5]. Thus, selecting the appropriate emulsifier is one of the key aspects in producing emulsion and dispersion systems. Polysorbates and sucrose esters are both nonionic emulsifiers that play important roles in various food products [6]. The polysorbates are produced by reacting approximately 20 moles of ethylene oxide with one molecular weight equivalent of sorbitan ester. Due to the presence of the resulting long polyoxyethylene chain, they are the most hydrophilic emulsifiers among the nonionic types [7,8]. Sucrose esters are synthesized by the esterification of fatty acids with sucrose, a polyhydric alcohol with eight hydroxyl groups that has three primary and five secondary hydroxyls; the primary ones are more reactive and more easily replaced with fatty acids. The distinctive characteristics of each polysorbate or sucrose ester are related to the various fatty acids used in their structures. Nutritionally, they are hydrolyzed by digestive enzymes into their component molecules that can then be metabolized in the usual way [4,8,9]. Being biodegradable, non-toxic, non-irritant to the skin, polysorbates and sucrose esters of fatty acid are human- and environmental-friendly [8]. The use of polysorbates and sucrose esters of fatty acids is therefore expected to increase greatly in the food, cosmetic and pharmaceutical industries in the near future.

Emulsifiers can stabilize emulsions and dispersions via electrostatic or/and steric mechanisms. Nonionic emulsifiers do not affect electrostatic stabilization, but they are very good steric stabilizers. In most cases, these emulsifiers are insensitive to the presence of any ions in solution, allowing for their wider application in various systems, especially in water of unknown hardness. The hydrophilic ends of these emulsifiers repel each other and thus provide stability to emulsions and dispersions [3,4,6,8].

In the past few years, nanotechnology has become one of the most interesting fields of scientific research. In the field of food science, a good understanding of the special properties of materials at the nanometer diameter will allow food scientists to design new, healthier, tastier and safer foods [10,11]. Improving the water solubility and consequently the bioavailability of functional lipid bioactive compounds of various foods such as carotenoids, phytosterols, polyunsaturated fatty acids and many other compounds is just some of the most important applications of nanotechnology in foods [7,10,11]. These functional lipid compounds have received increasing interest for their applications in the food, pharmaceutical and cosmetics industries in the last few years due to their enormous health benefits [1,4,11,12]. Most recently candesartan cilexetil and atorvastatin nanoparticles have been prepared using solvent evaporation techniques [13], while a simple homogenization process has been developed to produce cardanol-based micellar nanodispersions [14]. Tan and Nakajima [6] successfully prepared β-carotene nanodispersions using different polyglycerol esters of fatty acids as emulsifier. Yuan *et al.* [7] and Cheong *et al.* [15] have reported the characterization of β-carotene oil-in-water nanoemulsions and palm-based functional lipid nanodispersions, respectively, prepared by high pressure homogenization using a series of polyoxythylene sorbitan esters of fatty acids as emulsifiers. Leong *et al.* [16] have also studied the influence of different sucrose esters of fatty acids as emulsifiers on the physicochemical properties of phytosterol nanoemulsions. However, the effect of different polysorbates and sucrose esters of fatty acids have not been evaluated on physicochemical characteristics of astaxanthin nanodispersions.

Astaxanthin is a high-value, fat-soluble carotenoid that has been shown to exhibit high antioxidant capacity and to be a powerful scavenger of oxygen free radicals, a strong quencher of singlet oxygen activity, a cancer preventer and carcinogenesis inhibitor [12,17]. In a previous work the effect of emulsification and evaporation parameters on the physicochemical properties of astaxanthin nanodispersions was investigated, and the optimum processing conditions were obtained [12]. It was shown that the emulsification/solvent evaporation technique was applicable for the preparation of astaxanthin nanodispersions. However, in that study, Polysorbate 20 was the only small-molecule, nonionic emulsifier used to stabilize the nanodispersions. Thus, the objective of the current study was to investigate how the use of different nonionic emulsifiers affects the physicochemical properties of the resulting astaxanthin nanodispersions.

## 2. Results and Discussion

Emulsification-evaporation is one of the most suitable techniques for the preparation of carotenoid dispersions [11,12]. The influence of different emulsifier types is one of the important parameters that should be considered in nanodispersion preparation [7]. To investigate the influence of emulsifier type on the physicochemical properties of astaxanthin nanodispersions, all other parameters were fixed in the preparation of the different samples. Different types of emulsifiers exhibit different characteristics during homogenization: for example, the rate of adsorption onto the particles, maximum reduction in interfacial tension and the effectiveness of the interfacial membrane at preventing particle coalescence or oxidation, solubility in the aqueous phase and thermal stability [3].

### 2.1. Particle Diameter Parameters and Zeta-Potential of Astaxanthin Nanodispersions

Particle diameter and polydispersity index (PDI) are two of the most important parameters for determining the general quality of prepared astaxanthin nanodispersions. The type of attached monoester fatty acids and the hydrophilicities of the emulsifiers used are shown in Table 1.

**Table 1:** The type of attached monoester fatty acids and HLB values of the emulsifiers used.

Emulsifier	Combined fatty acid	HLB
Polysorbate 20	Lauric acid	16.7
Polysorbate 40	Palmitic acid	15.6
Polysorbate 60	Stearic acid	14.9
Polysorbate 80	Oleic acid	15
L-1695	Lauric acid (95%)	16
P-1570	Palmitic acid (70%)	15
S-1570	Stearic acid (70%)	15
OWA-1570	Oleic acid (70%)	15

The hydrophilicity of an emulsifier is measured by its hydrophilic-lipophilic balance (HLB) value; a higher HLB values indicate a higher hydrophilicity [3,6]. Emulsifiers with HLB values of 3.5 to 6 tend to be the most suitable for water in oil (W/O) emulsions, and those with HLB values from 8–18 are best suited for oil in water (O/W) emulsions [3,8]. Most commercial emulsifiers, especially sucrose esters, are mixtures of mono-, di- and triesters. Emulsifier HLB values are largely dependent on the monoester content; a greater monoester content leads to a higher HLB value [8]. The HLB value also depends on the chain length(s) of the fatty acids attached to the emulsifier; short fatty acid chains lead to higher HLB values [6,7]. 

The particle diameters and PDI values of the nanodispersions prepared with the different emulsifiers at constant concentrations are shown in Table 2. The particle diameter distributions changed from unimodal (e.g., Polysorbate 20), to multimodal (e.g., S-1570). The mean particle diameter of the resulting nanodispersions ranged between 70 and 170 nm. Therefore, the results confirmed that using nonionic emulsifiers can produce astaxanthin nanodispersions with the particle diameters in the nanometer range. Among the four different polysorbate emulsifiers used, Polysorbate 20 produced the smallest particles. Similarly, Cheong *et al.* [13] showed that palm-based functional lipid nanodispersions had the smallest particle size if stabilized by Polysorbate 20 as compared to other polysorbates. The particle diameters of the nanodispersions prepared with the other three polysorbate emulsifiers increased as their HLB decreased and their combined fatty acid chain lengths increased. These results are consistent with the previous results obtained by Tan and Nakajima [6] using polyglycerol esters of fatty acids in the production of β-carotene nanodispersions. No significant difference was observed with the Polysorbate 60 and Polysorbate 80 nanodispersions due to their relatively similar HLB and attached fatty acid lengths. As reported by Tan and Nakajima [6], emulsifiers with greater HLB can stabilize the particles in an O/W emulsion more efficiently and rapidly, thus resulting in smaller particles. It may be related to this fact that the surface area of nanoparticles stabilized by an emulsifier increases with the increasing hydrophilicity of the emulsifier, resulting in smaller particles that have better covering, wrapping and stabilizing efficiencies [4,6,7]. It should be noted that the 20 moles of oxyethylene used in polysorbate emulsifiers is the minimum required for effective stabilization. Longer chain products with 50–100 oxyethylene molecules are more effective emulsion stabilizers but are not allowed in food products [8]. Among sucrose ester emulsifiers, the particle diameter of nanodispersions produced by L-1695 was less than that with P-1570, and both were less than those produced by S-1570 due to its shorter attached fatty acids and higher HLB values (in the case of L-1695). However, the smaller particle diameter of astaxanthin nanodispersions stabilized by OWA-1570 (having the same fatty acid chain length and HLB as S-1570) may be related to the initial formulation of OWA-1570 in its manufacture; it is a mixture of 40% O-1570, 4% ethanol and 56% water. Including the ethanol in this emulsifier formulation may facilitate solvent diffusion into the water phase, providing a greater driving force for evaporation at the air-liquid interface, resulting in the significant decrease in the diameter of the crystallized or precipitated particles produced during the evaporation process [4,18]. These results disagreed with those of Leong *et al.* [17] who found that the particle diameter of OWA-1570 stabilized phytosterol nanoemulsions are larger than other sucrose esters-stabilized ones and also there are no significant (*p* > 0.05) differences among produced astaxanthin nanodispersions using L-1695, P-1570 and S-1570 with the concentration of 1% as stabilizer in term of particle diameters. The differences between these two studies can be related to the dissimilarities of studied systems such as the physical state, nature and concentrations of active compounds. 

**Table 2:** Average particle size (nm), PDI and zeta potential of astaxanthin nanodispersions prepared with different emulsifier.

Emulsifier	Particle size (nm)	PDI	Zeta Potential (mV)
Polysorbates			
Polysorbate 20	75.0 ± 3.2 ^d^	0.376 ± 0.023 ^cd^	−14.1 ± 0.6 ^c^
Polysorbate 40	83.5 ± 2.6 ^bc^	0.642 ± 0.037 ^a^	−23.3 ± 2.5 ^b^
Polysorbate 60	139.7 ± 7.2 ^a^	0.541 ± 0.093 ^ab^	−22.8 ± 2.6 ^b^
Polysorbate 80	160.3 ± 10.0 ^a^	0.474 ± 0.092 ^bc^	−24.3 ± 4.9 ^b^
Sucrose esters			
L-1695	73.1 ± 2.2 ^d^	0.242 ± 0.030 ^e^	−19.2 ± 1.2 ^b^
P-1570	85.2 ± 2.0 ^b^	0.281 ± 0.022 ^e^	−21.2 ± 2.0 ^b^
S-1570	143.5 ± 7.4 ^a^	0.424 ± 0.062 ^bc^	−30.0 ± 2.2 ^a^
OWA-1570	79.8 ±1.2 ^c^	0.355 ± 0.005 ^d^	−21.0 ± 2.7 ^b^

Values are means ± standard deviations (*n* = 6); ^a–c^ Different letters show statistically significant differences between treatments (*p* < 0.05).

The PDI values show the width of the particle diameter distribution; here, a small PDI value indicates a narrow particle diameter distribution and *vice versa* [19]. As shown in Table 2, in general, all the emulsifiers used exhibited an acceptable range of size distributions (PDI < 0.5), but the astaxanthin nanodispersions produced with sucrose esters had lower PDI values than the polysorbate-stabilized nanodispersions. This can be attributed to the higher entrapment efficiency of sucrose esters as well as their higher critical micelle concentrations than polysorbates, which increase the production of astaxanthin loaded nanodispersions and decrease the production rate of emulsifier micelles. Therefore, the PDI values of sucrose ester stabilized nanodispersions was smaller as compared to polysorbates stabilized ones [3,17]. 

**Figure 1:**
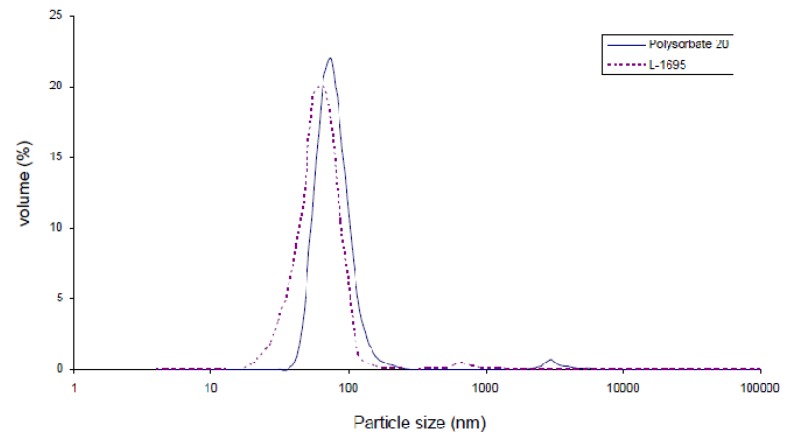
Particle size distribution of astaxanthin nanodispersions prepared with Polysorbate 20 and L-1695.

Polysorbate 40 and 60 and S-1570 produced nanodispersions with slightly higher PDI values than the others. The above results (Table 2) showed that Polysorbate 20 and L-1695 produced the astaxanthin nanodispersions with smallest particle diameters and narrowest size distributions among the polysorbate and sucrose ester emulsifiers, respectively (Figure 1). 

As shown in Table 2, all the astaxanthin nanodispersions produced had a negative net surface charge. Except for Polysorbate 20 and S-1570, which produced the nanodispersions with the minimum and maximum negative net surface charges, respectively, no significant differences (*p* < 0.05) were seen in the zeta potentials of the nanodispersions stabilized with the other emulsifiers. The negative surface charge of the nanodispersions may be due to the adsorption of OH- species from the aqueous phase or cationic impurities from the oil/carotenoid mixture onto the droplet interface [3,9]. As stated above, the predominant stabilizing mechanism of nonionic emulsions such as polysorbates and sucrose esters is steric hindrance. However, the existence of the ions on the particle surfaces can also aid in stabilizing the system via electrostatic repulsion. Thus, a higher net surface charge (*i.e*., a higher net zeta potential) can provide a more stable emulsion or dispersion system [3]. 

### 2.2. Astaxanthin Loss of Astaxanthin Nanodispersions

Carotenoid molecules are very sensitive to light, oxygen and heat and may easily undergo auto-oxidation and undesired reactions in these severe preparation and storage conditions [11,12]. As stated by Tan and Nakajima [11], the degradation of carotenoids in a nanoemulsion or nanodispersion occurs for two major reasons: the large surface area of the particles as a result of their diameter reduction to the nanometer range and the creation of free radicals during the high-pressure homogenization process. Small particles have a high surface area and thus increased exposure to light, free radicals, *etc.*, which can cause high astaxanthin loss in nanodispersions. The possible temperature rise in dynamic high-energy systems may increase the probability of free radical production in these nanosize systems. The astaxanthin contents of the freshly prepared coarse emulsions with different types of emulsifiers after convention homogenization and also after the solvent evaporation step (final product) are shown in Table 3. 

**Table 3:** Astaxanthin concentration of nanodispersions during preparation steps.

Emulsifier	Astaxanthin concentration (mg/L)		Astaxanthin loss (% w/w)
	After convention homogenization (mg/L)	After evaporation (mg/L)	During high pressure homogenizer and evaporation processes
Polysorbate 20	710.7 ± 6.4 ^a^	593.7 ± 3.4 ^e^	15.98 ± 0.74 ^B^
Polysorbate 40	704.6 ± 3.5 ^a^	601.0 ± 7.0 ^e^	14.94 ± 1.14 ^B^
Polysorbate 60	702.1 ± 5.4 ^a^	596.1 ± 3.0 ^e^	15.64 ± 0.70 ^B^
Polysorbate 80	706.5 ± 3.3 ^a^	580.2 ± 2.2 ^f^	17.89 ± 0.64 ^A^
L-1695	709.7 ± 5.1 ^a^	601.1 ± 5.5 ^e^	14.93 ± 0.96 ^B^
P-1570	710.9 ± 5.9 ^a^	616.3 ± 1.8 ^c^	12.78 ± 0.61 ^C^
S-1570	706.8 ± 2.9 ^a^	640.0 ± 2.5 ^b^	9.43 ± 0.66 ^D^
OWA-1570	701.0 ± 4.8 ^a^	580.4 ± 3.2 ^f^	17.86 ± 0.72 ^A^

Values are means ± standard deviations (*n* = 6); ^a−f^ Different letters show statistically significant differences between astaxanthin concentration values of treatments (*p* < 0.05); ^A−D^ Different letters show statistically significant differences between astaxanthin loss values of treatments (*p* < 0.05).

As shown here, the astaxanthin concentration of all prepared samples with different emulsifiers was equal and approximately 706.6 ± 4.7 mg/L after convention homogenization process. After the high-pressure homogenization and solvent evaporation steps, astaxanthin loss occurred to different degrees depending on the type of emulsifier used. The results showed that, among the various prepared nanodispersions, astaxanthin degradation was considerably higher in the Polysorbate 80- and OWA-1570-stabilized preparations than in the others. The presence of an unsaturated fatty acid (oleic acid) in these emulsifiers’ structure could be the reason for the noticeably lower astaxanthin concentrations observed in these final dispersion products because double bonds can increase the oxidation rate of astaxanthin [6]. The higher astaxanthin loss in the polysorbate-stabilized nanodispersions compare with those stabilized with sucrose esters could be due to reduced formation of protective barriers on the astaxanthin nanoparticles by these emulsifiers. Among all the nanodispersions, those stabilized with S-1570 showed the highest astaxanthin content and consequently the most chemical stability. The astaxanthin losses for the Polysorbate 20- and L-1695-stabilized nanoparticles did not differ significantly (*p* < 0.05). Therefore, these emulsifiers provide the same chemical stability for astaxanthin nanoparticles in the studied dispersion systems. 

## 3. Experimental

### 3.1. Materials

Synthetic astaxanthin (>90%) was purchased from Kailu Ever Brilliance Biotechnology Co., Ltd. (Beijing, China). Sucrose laurate (L-1695), sucrose palmitate (P-1570), sucrose stearate (S-1570) and sucrose oleate (OWA-1570) were donated by Mitsubishi Food Co. (Tokyo, Japan). Polyoxyethylene sorbitan monolaurate (Polysorbate 20), polyoxyethylene sorbitan monopalmitate (Polysorbate 40), polyoxyethylene sorbitan monostearate (Polysorbate 60), polyoxyethylene sorbitan monooleate (Polysorbate 80), sodium azide, analytical and HPLC-grade dichloromethane, methanol and acetonitrile were provided by Fisher Scientific (Leicestershire, UK). 

### 3.2. Preparation of Astaxanthin Nanodispersions

The production of astaxanthin nanodispersions via the emulsification-evaporation method consists of two main steps: First, the preparation of a solvent-in-water emulsion and then its conversion into nanodispersion by removing the solvent. Here, various types of emulsifiers were dissolved in deionized water (1% w/w) containing sodium azide (0.02% w/w) under magnetic stirring to produce the aqueous phase. Next, an organic phase consisting of astaxanthin (1% w/w) dissolved in dichloromethane was added to the aqueous phase at the organic:aqueous ratio of 1:9 by weight. The mixture was then homogenized using a conventional homogenizer (Silverson, L4R, Buckinghamshire, UK) at 5,000 rpm for 5 min. The initial emulsion produced was then passed through a high-pressure homogenizer (APV, Crawley, UK) at 50 MPa for two cycles. The solvent was then removed from the fine emulsion by rotary evaporation (Eyela NE-1001, Tokya Rikakikai Co. Ltd., Tokyo, Japan) at 250 Pa and 47 °C [12]. The formation of astaxanthin particles occurs by diffusion of the organic phase into the aqueous phase and evaporation at the air/water interface. The hydrophilic segments of the emulsifier molecules extend into the water phase to form a viscous layer, inhibiting the aggregation of astaxanthin particles [6,17].

### 3.3. Analytical Methods

#### 3.3.1. Mean Particle Diameter and Polydispersity Index (PDI)

Measurement of the mean particle diameter (D_43_) of the nanodispersions and their polydispersity index was performed with a series ZEN 1600 dynamic light scattering particle analyzer (Malvern Instruments Ltd., Worcester, UK). The experiments were performed with samples diluted (1:10) with deionized water to avoid multiple scattering effects in the measurements. The absorbance of the nanodispersion particles was set at 0.3, and the temperature was 25 °C. A laser beam was passed through the samples and scattered by the particles; the scattered light was then detected by an array of photodiodes placed behind the cuvette. The final particle diameter was calculated from an average of three measurements [12]. 

#### 3.3.2. Zeta-Potential Measurement

The zeta-potential values of the astaxanthin nanodispersions were measured using a Zetasizer Nano ZS (Malvern Instruments Ltd). The distribution of the electrophoretic mobility of particles is measured based on laser Doppler velocity technique. The Smoluchowski equation was employed to calculate the zeta potential values from the measured velocity [9]. Measurements were performed at pH 7.

#### 3.3.3. Determination of Astaxanthin Content

##### 3.3.3.1. Sample Preparation for Astaxanthin Determination

The sample preparation method for astaxanthin analysis was modified from Higuera-Ciapara’s work [20]. A sample aliquot (0.5 mL) was added to a mixture of dichloromethane and methanol (50:50 v/v, 2 mL) in an amber vial with a screw top. The vial was closed tightly and agitated for 15 min. The mixture was centrifuged at 800 ×*g* for 5 min using a Kobota 2010 (Tokyo, Japan) centrifuge at room temperature. The extract was then decanted. This extraction procedure was repeated two more times. The volume of sample was brought up to 10 mL by the addition of methanol. A sample aliquot was filtered with a membrane filter, and 40 μL of filtrate was injected into the HPLC.

##### 3.3.3.2. HPLC Analysis

HPLC analysis was performed using an Agilent 1200 Series liquid chromatography system (Agilent Technologies, Waldbron, Germany), equipped with a G13150 Diode Array Detector and a Waters Nova-Pak^®^ C18 (3.9 × 300 mm) HPLC column, using an isocratic mobile phase consisting of 85% methanol, 5% dichloromethane, 5% acetonitrile and 5% water. The detection was performed at 480 nm [17,21]. The calibration curve of peak area *versus* astaxanthin concentration was linear in the range of measured concentrations (R^2^ = 0.9916, n = 4).

### 3.4. Statistical Analysis

The physicochemical properties of the astaxanthin nanodispersions were subjected to one-way analysis of variance (ANOVA) using the Minitab v. 14 statistical package (Minitab Inc., University Park, PA, USA). All experiments and measurements were performed in duplicate. Significantdifferences (*p* < 0.05) between means were determined by Tukey’s multiple range tests.

## 4. Conclusions

The performance of selected polysorbate and sucrose ester emulsifiers in the preparation of astaxanthin nanodispersions was investigated in this work. The results showed that the type of emulsifier used affected the physicochemical properties of the resulting astaxanthin nanodispersions. In summary, emulsifiers with higher HLB values (higher hydrophilicities) and shorter fatty acid chains produced nanodispersions with smaller particle diameters. The results also showed that the presence of unsaturated fatty acids in the emulsifier structure led to considerably increased astaxanthin loss in the nanodispersions produced. For this technology to be applied commercially, further research should be done to study and improve the physicochemical stability of astaxanthin during the nanodispersion production process and its subsequent storage under varying storage conditions. 
